# Metabolic Changes during In Vivo Maturation of PSC-Derived Skeletal Myogenic Progenitors

**DOI:** 10.3390/cells13010076

**Published:** 2023-12-29

**Authors:** Phablo Abreu, Bayardo I. Garay, Travis Nemkov, Aline M. S. Yamashita, Rita C. R. Perlingeiro

**Affiliations:** 1 Lillehei Heart Institute, Department of Medicine, University of Minnesota, Minneapolis, MN 55455, USA; abreu030@umn.edu (P.A.); garay034@umn.edu (B.I.G.); sakag012@umn.edu (A.M.S.Y.); 2 Department of Neuroscience, University of Minnesota, Minneapolis, MN 55455, USA; 3 Department of Biochemistry and Molecular Genetics, University of Colorado Anschutz Medical Campus, Aurora, CO 80045, USA; travis.nemkov@cuanschutz.edu; 4 Stem Cell Institute, University of Minnesota, Minneapolis, MN 55455, USA

**Keywords:** pluripotent stem cells, myogenic progenitors, metabolism, in vitro, in vivo, satellite cells

## Abstract

In vitro-generated pluripotent stem cell (PSC)-derived Pax3-induced (iPax3) myogenic progenitors display an embryonic transcriptional signature, but upon engraftment, the profile of re-isolated iPax3 donor-derived satellite cells changes toward similarity with postnatal satellite cells, suggesting that engrafted PSC-derived myogenic cells remodel their transcriptional signature upon interaction within the adult muscle environment. Here, we show that engrafted myogenic progenitors also remodel their metabolic state. Assessment of oxygen consumption revealed that exposure to the adult muscle environment promotes overt changes in mitochondrial bioenergetics, as shown by the substantial suppression of energy requirements in re-isolated iPax3 donor-derived satellite cells compared to their in vitro-generated progenitors. Mass spectrometry-based metabolomic profiling further confirmed the relationship of engrafted iPax3 donor-derived cells to adult satellite cells. The fact that in vitro-generated myogenic progenitors remodel their bioenergetic signature upon in vivo exposure to the adult muscle environment may have important implications for therapeutic applications.

## 1. Introduction

Homeostasis of the skeletal muscle is provided by muscle stem cells, also known as satellite cells. Upon injury, satellite cells become activated and give rise to proliferating myoblasts, which will differentiate into new myofibers and/or fuse to existing muscle fibers or to other myoblasts to repair muscle damage [1,2,3]. A small subset of satellite cells retain the ability to self-renew, thus preserving the satellite cell reservoir [3,4]. While satellite cells have remarkable regenerative potential, their application on cell-based therapies for skeletal muscle disorders remains challenging due to limitations associated with ex vivo expansion [2]. This is required since the number of satellite cells in a small muscle biopsy is not sufficient for cell-based therapy. A major caveat with ex vivo expansion is that once removed from the muscle environment and allowed to expand, satellite cells differentiate into myoblasts, which have limited regenerative potential [2,5,6]. Because pluripotent stem cells (PSCs) can repeatedly produce large amounts of lineage-specific cell types, they represent an attractive source for the generation of muscle stem/early progenitor cells for therapeutic applications. We have reported that the conditional expression of the transcription factors Pax3 (iPax3) or Pax7 (iPax7) in differentiating PSCs enables the generation of large numbers of myogenic progenitors endowed with in vivo regenerative potential [7,8,9,10,11]. Of interest is the fact that, while in vitro-generated PSC-derived myogenic progenitors display an embryonic transcriptional profile, this changes upon transplantation as engrafted re-isolated donor-derived satellite cells show a postnatal molecular signature [12]. The majority of the genes found to be differentially expressed between in vitro-generated PSC-derived myogenic progenitors and adult bona fide satellite cells were no longer distinct in re-isolated donor-derived cells, including genes of the Notch pathway [12], known to be important for satellite cell quiescence and function [13,14,15].

Metabolic pathways have also been reported to play an important role in satellite cell function during development and adult homeostasis, as well as upon stress [16,17,18,19]. However, the metabolic profile of in vitro-generated PSC-derived myogenic progenitors and subsequent post-transplant donor-derived satellite cells has never been investigated.

To determine the metabolic status of PSC-derived myogenic progenitors and potential changes upon their in vivo exposure to the adult muscle environment, here we assessed transcriptional profiles pertinent to the metabolic state using published datasets [12] and performed real-time metabolic flux analysis and mass spectrometry-based metabolomics in in vitro-generated iPax3 myogenic progenitors and post-transplant iPax3 donor-derived satellite cells side by side with adult satellite cells. Our results show stark changes in all parameters when comparing in vitro-generated myogenic progenitors to their in vivo post-transplant re-isolated counterparts and demonstrate that iPax3 donor-derived satellite cells assume a metabolic profile similar to bona fide satellite cells.

## 2. Material and Methods

### 2.1. RNA-Sequencing and Microarray Analysis

Bulk RNA-sequencing datasets from single-end libraries using the Illumina platform were downloaded from the gene expression omnibus (GEO) database using the Sequence Read Archive (SRA) toolkit for 3 samples of mouse PSC-derived iPax3 myogenic progenitors and 3 samples of adult satellite cells [12]. Additionally, Whole Mouse Genome Microarray 4x44K G4122F datasets for 3 samples of post-transplant re-isolated iPax3 donor-derived mononuclear cells (GFP+CD34+) and 3 samples of adult satellite cells [12] were downloaded using the GEOQuery [20] package (v2.66.0) on R. Data alignment and comparison of expression were analyzed using the CHURP pipeline [21] at the University of Minnesota Genomics Center (UMGC). We trimmed 1 × 100 bp FASTQ single-end reads for 6 samples (34.2 million reads average per sample) using Trimmomatic (v0.33) with the optional “-q” option enabled; 3 bp sliding-window trimming from 3′ end requiring minimum Q30. We performed quality control on raw sequence data for each sample with FastQC and read mapping with HISAT2 [22] (v2.1.0) using the mouse genome (GRCh39.106) as reference. We performed gene quantification using Feature Counts for raw read counts. We identified differentially expressed genes using the EdgeR (negative binomial, R programming) feature in CLCGWB (Qiagen, Redwood City, CA, USA) using raw read counts. We filtered the generated list based on a minimum 2× absolute fold change and FDR-corrected *p* < 0.05. GSE analysis using the clusterProfiler [23,24] package (v4.6.0) on R was used to carry out Reactome [25] and KEGG [26] pathway analysis and gene ontology enrichment analysis. Pathways with an FDR *q* < 0.05 were considered to be significantly expressed. For the microarray data, pairwise comparisons, at the gene-level, were conducted using the limma [27] package on R (v3.54.0).

### 2.2. Cell Culture

For the studies described here, we used H2B-GFP-labelled inducible Pax3 (iPax3) embryonic stem (ES) cells, which were differentiated as before [12]. Briefly, ES cells were maintained in a combination of ES medium and 2 inhibitors (2i) medium (1:1). ES medium consists of KnockOut^TM^ DMEM (Invitrogen, Waltham, MA, USA) containing 15% FBS (Sigma, Burlington, MA, USA), 1% penicillin-streptomycin (Invitrogen), 2 mM Glutamax (Gibco, Waltham, MA, USA), 0.1 mM non-essential amino acids (Gibco), and 0.1 mM β-mercaptoethanol (Gibco), whereas the 2i medium consists of neurobasal medium (Invitrogen) and DMEM F12 medium (Invitrogen) containing 0.5% N2 (Life Technologies, Carlsbad, CA, USA), 0.5% B27 (Life Technologies), 0.05% BSA (Sigma), 1% penicillin-streptomycin, 150 µM monothioglycerol (MP Biomedicals, Santa Ana, CA, USA), 3 µM GSK3β inhibitor (CHIR 990217; Tocris, Minneapolis, MN, USA), 1 µM PD 0325901 (Cayman, Ann Arbor, MI, USA), and 1000 U/mL LIF (Millipore, Burlington, MA, USA). For differentiation into embryoid bodies (EB), we cultured ES cells in suspension at the concentration of 40,000 cells/mL in culture medium containing IMDM (Invitrogen), 15% FBS (Sigma), 1% penicillin/streptomycin (Invitrogen), 2 mM GlutaMAX (Invitrogen), 50 μg/mL ascorbic acid (Sigma-Aldrich, Burlington, MA, USA), and 4.5 mM monothioglycerol (MP biomedicals). We induced Pax3 expression by adding doxycycline (dox; Sigma-Aldrich D989) to cultures at day 3 of EB differentiation (final concentration 1 μg/mL). At day 5, EBs were sorted for the Vcam1+FLK1- cell fraction [12]. Briefly, EBs were disaggregated, incubated with Fc block (1 μL/million cells; BD Biosciences, Franklin Lakes, NJ, USA) for 5 min, and then stained with Flk1-APC and Vcam1-biotin-conjugated antibodies (1 μL/million cells; e-Bioscience) for 60 min on ice**,** followed by 20 min incubation with streptavidin-PeCy7. Cells were washed twice with PBS and then resuspended in PBS containing 10% FBS and propidium iodide (PI) to exclude dead cells. Vcam1+FLK1- cells were sorted using a FACSAria II (BD Biosciences), plated on gelatin-coated dishes in the presence of EB differentiation media containing 1 μg/mL dox and 10 ng/mL mouse basic FGF (bFGF; PeproTech, Rocky Hill, NJ, USA; #100-18). After 3 passages, cells were used for transplantation studies. 

### 2.3. Mice and Cell Transplantation

All animal experiments were carried out in strict accordance with protocols approved by the University of Minnesota Institutional Animal Care and Use Committee. Twelve-week-old male Pax7-ZsGreen reporter mice [28] and six–eight-week-old male NOD-scid IL2Rg^null^ (NSG) mice (Jackson Laboratories, Bar Harbor, ME, USA) were used for satellite cell isolation (reference group) and transplantation studies, respectively. As previously described [12], 2 days prior to intramuscular transplantation, both hind limbs were subjected to a 12 Gy dose of local irradiation using an RS 2000 Biological Research Irradiator (Rad Source Technologies, Inc., Buford, GA, USA). One day before cell transplantation, we injured both TA muscles with 15 μL of cardiotoxin (10 μM in PBS, Sigma, Saint Louis, MO, USA). The next day, 3 × 10^5^ iPax3 myogenic progenitors were transplanted into injured muscles. Four weeks later, mice were euthanized for assessment of skeletal muscle engraftment and re-isolation of donor-derived satellite cells.

### 2.4. Satellite Cell Purification

We purified post-transplant donor-derived satellite cells based on the expression of GFP (donor tracking) and the following satellite cell marker profile: CD31−/CD45−/Itga7+/CD34+ [29]. For primary adult satellite cells, the ZsGreen+/CD31−/CD45−/Itga7+/CD34+ cell fraction was sorted. 

### 2.5. Metabolic Flux Analysis

Oxygen consumption rates (OCR) and extracellular acidification rates (ECAR) of iPax3 myogenic progenitors and donor-derived satellite cells, as well as bona fide satellite cells, were measured using the Seahorse XF96 Analyzer (Seahorse Bioscience, Billerica, MA, USA), as previously described [18]. Cells were seeded onto matrigel-coated XF96 96-well microplates (for 24 h) at 10,000/well for in vitro iPax3 cells and re-isolated in vivo iPax3 cells and at 70,000/well for adult satellite cells. Following titration, optimal concentrations of respiratory modulators were chosen for these studies. Prior to the assay, we replaced the medium with 180 μL of 5 mM glucose-containing medium without sodium bicarbonate. Cells were pre-incubated for 1 h at 37 °C and 0% CO_2_ prior to loading into a Seahorse Bioscience XF96 extracellular flux analyzer. Over approximately 90 min, the ports of the cartridge containing the oxygen probes were loaded with the compounds to be injected during the assay and the cartridge was calibrated. ATP synthesis-linked O_2_ consumption and proton leak-driven respiration were determined through the addition of oligomycin (1 μg/mL, Sigma, # O4876). After 3 measurement cycles, the uncoupler carbonyl cyanide 3-chlorophenylhydrazone (2 μM CCCP, Sigma, # C2759) was added to promote maximal respiratory capacity. After 3 additional measurement cycles, we added rotenone (1 μM, Sigma, R8875) and antimycin A (1 μg/mL, Sigma, A8674) to inhibit complex I and complex III, respectively, therefore resulting in depletion of mitochondrial oxygen consumption.

### 2.6. qRT-PCR

Cells were harvested using TRIzolTM (Invitrogen). RNA was extracted using the PurelinkTM RNA Mini kit (Invitrogen) and reverse transcribed using the Superscript^®^ VILOTM cDNA synthesis kit (Invitrogen). TaqMan probes for *Pax7*, *Sdhd*, *Cox6b2*, *Atp5c1*, and *Gapdh* were used (Applied Biosystems, Waltham, MA, USA).

### 2.7. Immunofluorescence

TA muscles were embedded in Tissue-Tek O.C.T. (Sakura, Finetek Japan. Co., Ltd., Tokyo, Japan) and snap frozen on isopentane pre-cooled with liquid nitrogen. Cryosections of 14 µm were collected on glass slides and preserved at −80 °C. Prior to staining, muscles cryosections were rehydrated with PBS for 5 min at room temperature (RT), fixed with 4% PFA for 30 min at RT, washed with PBS, permeabilized 15 min at RT with 0.3% Triton X100 (Sigma) in PBS, washed again with PBS, blocked for 30 min with 3% BSA (Sigma), and incubated overnight at 4 °C with primary antibodies for GFP (chicken 1:500; Abcam, Waltham, MA, USA; #13970) and dystrophin (mouse 1:20; Leica, Nanterre Cedex, France; DYS1-CE). The next day, cryosections were rinsed with PBS and incubated with goat anti-chicken (1:500; Thermo Fisher, # A-11039) and goat anti-mouse (1:500; Thermo Fisher; #A-21235) secondary antibodies for 1 h at RT. Following three PBS washes, slides were dried and mounted with Prolong Gold with DAPI (Invitrogen). 

### 2.8. Metabolomics Sample Preparation and UHPLC-MS Analysis

In preparation for sample submission, cells were counted, pelleted, and stored at −80 °C. Prior to LC-MS analysis, samples were placed on ice and re-suspended with methanol:acetonitrile:water (5:3:2) at a concentration of 2 million cells per ml. Suspensions were vortexed continuously for 30 min at 4 °C. Insoluble material was removed via centrifugation at 10,000× *g* for 10 min at 4 °C. Isolated supernatants were dried under vacuum and resuspended in 15% methanol containing 0.1% formic acid.

UHPLC-MS analyses were performed as previously described [30,31]. Briefly, the analytical platform employs a Vanquish UHPLC system (Thermo Fisher Scientific, San Jose, CA, USA) coupled online to a Q Exactive mass spectrometer (Thermo Fisher Scientific). Polar extracts (2 µL injections) were resolved over a Kinetex C18 column, 2.1 × 150 mm, 1.7 µm particle size (Phenomenex, Torrance, CA, USA), equipped with a guard column (SecurityGuard^TM^ Ultracartridge—UHPLC C18 for 2.1 mm ID Columns—AJO-8782—Phenomenex, Torrance, CA, USA) using an aqueous phase (A) of water and 0.1% formic acid and a mobile phase (B) of acetonitrile and 0.1% formic acid for positive ion polarity mode, and an aqueous phase (A) of water:acetonitrile (95:5) with 1 mM ammonium acetate and a mobile phase (B) of acetonitrile:water (95:5) with 1 mM ammonium acetate for negative ion polarity mode. The Q Exactive mass spectrometer (Thermo Fisher Scientific) was operated independently in positive or negative ion mode, scanning in Full MS mode (2 μscans) from 60 to 900 *m/z* at 70,000 resolution, with 4 kV spray voltage, 45 sheath gas, 15 auxiliary gas, AGC target = 3 × 10^6^, and maximum IT = 200 ms. Samples were analyzed in randomized order with a technical mixture injected after every 10 samples to qualify instrument performance. Calibration was performed prior to analysis using the Pierce^TM^ Positive and Negative Ion Calibration Solutions (Thermo Fisher Scientific). Acquired data were converted from raw to mzXML file format using Mass Matrix (Cleveland, OH, USA). Metabolite assignments were performed using accurate intact mass (sub-10 ppm), isotopologue distributions, and retention time/spectral comparison to an in-house standard compound library (MSMLS, IROA Technologies, NJ, USA) using MAVEN (Princeton, NJ, USA). 

### 2.9. Statistical Analysis

GraphPad Prism v9 (GraphPad Software, LLC) was used to perform one-way and two-way ANOVA with post hoc correction for multiple comparisons using the Bonferroni–Holm or Tukey tests. *p* values < 0.05 were considered statistically significant. Multivariate analyses of metabolomics data including principal component analysis, hierarchical clustering analysis, ANOVA, and pathway enrichment analysis were performed using Metaboanalyst 5.0 [32].

## 3. Results

### 3.1. Transcriptional Landscape of Metabolic Pathways in iPax3 Myogenic Progenitors

**Figure 1:**
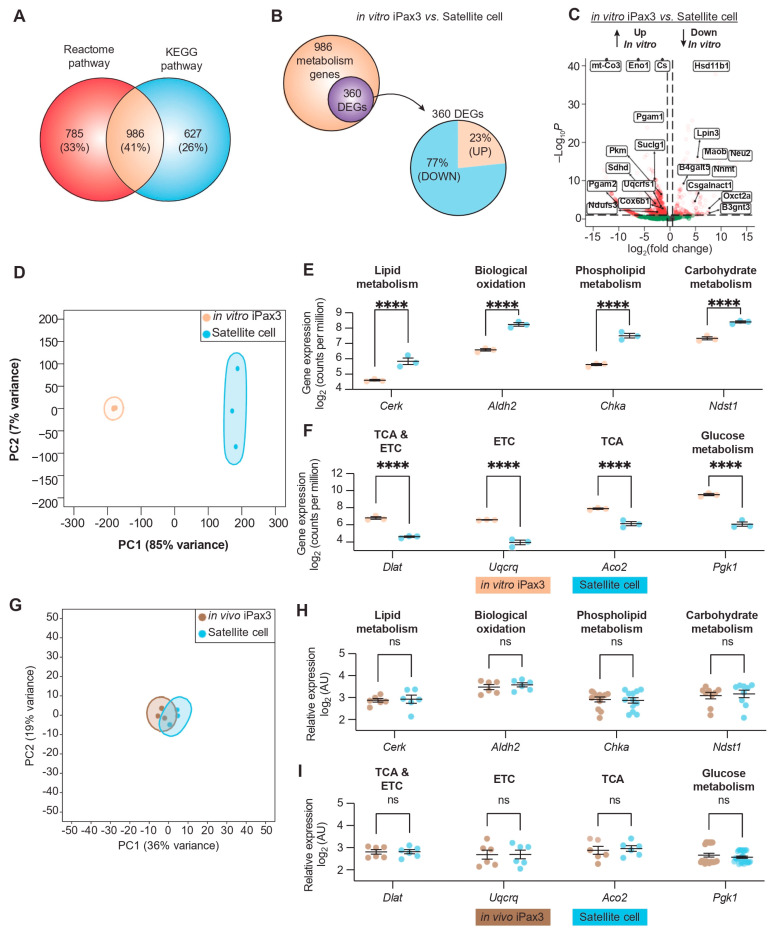
Transcriptional profile of metabolic programs in murine iPax3 myogenic progenitors compared to adult satellite cells. (**A**) Venn diagram of genes annotated to metabolic pathways with the Reactome and KEGG databases. (**B**) Venn diagram showing overlap between differentially expressed genes (DEGs) after pairwise comparisons and genes annotated to metabolic pathways (top), as well as a breakdown of upregulated (brown) and downregulated (blue) DEGs (bottom). (**C**) Volcano plot for 986 metabolic genes. (**D**) Principal component analysis (PCA) of bulk RNA-seq in in vitro iPax3 (orange) and satellite cells (blue). (**E**,**F**) Representative gene expression levels of in vitro iPax3 myogenic progenitors (orange) vs. satellite cells (blue) across metabolic pathways enriched in cluster 1 (**E**) and cluster 2 (**F**). (**G**) PCA of microarray in in vivo iPax3 myogenic progenitors (brown) and adult satellite cells (blue). (**H**,**I**) Representative gene expression levels of in vivo iPax3 myogenic progenitors (brown) vs. satellite cells (blue) across metabolic pathways previously enriched in cluster 1 (**H**) and cluster 2 (**I**). Studies represent three biological samples per group. **** *p* < 0.0001 by ANOVA with Bonferroni correction. Abbreviations: Tricarboxylic acid (TCA), Electron Transport Chain (ETC).

To determine the transcriptional profile pertinent to the metabolic state of in vitro-generated PSC-derived Pax3-induced (iPax3) myogenic progenitors in comparison to adult Pax7^+^ satellite cells, we interrogated metabolic genes in our published datasets [12]. By combining the annotations from the Reactome pathway [25] and the Kyoto Encyclopedia of Genes and Genomes (KEGG) pathway [26] databases, we identified 986 shared genes related to metabolic pathways (Figure 1A). Pairwise comparisons between in vitro-generated iPax3 myogenic progenitors (hereafter referred to as in vitro iPax3) and adult satellite cells revealed 360 differentially expressed genes (DEGs) mapping onto metabolic pathways, with the majority (77%) of the DEGs being significantly underexpressed in in vitro iPax3 cells compared to adult satellite cells (Figure 1B). This analysis showed that in vitro iPax3 cells display significant overexpression of genes involved in mitochondrial respiration, such as mitochondrial-encoded cytochrome C oxidase III (*mt-Co3*), mitochondrial membrane ATP synthase (*Atp5pb*), and succinate dehydrogenase complex (*Sdhd*), relative to adult satellite cells (Figure 1C). In contrast, they display significantly lower expression of genes involved in fatty acid and lipid metabolism, such as lipin 3 (*Lpin3*), hydroxysteroid 11-β dehydrogenase (*Hsd11b1*), and neuraminidase 2 (*Neu2*), relative to adult satellite cells (Figure 1C). Linear dimensionality reduction via principal component analysis (PCA) unveiled stark differences between in vitro iPax3 cells and adult satellite cells (Figure 1D). Unsupervised hierarchical clustering analysis of the 360 metabolic DEGs revealed two main clusters (Supplementary Figure S1A). Gene ontology (GO) analysis of these two clusters showed highly enriched genes related to lipid metabolic process, carbohydrate derivative metabolic process, and membrane lipid metabolic process (cluster 1, Supplementary Figure S1B) as well as oxidation-reduction process, tricarboxylic acid (TCA) metabolic process, and respiratory electron transport chain (cluster 2, Supplementary Figure S1C).

In agreement with previous observations of freshly isolated quiescent satellite cells [33], our analysis of Pax7^+^ satellite cells revealed significant high expression of signaling pathways involved in lipid-, phospholipid-, and carbohydrate-metabolism (Figure 1E and Supplementary Figure S1D). On the other hand, in vitro iPax3 cells display enhanced expression of signaling pathways related to glucose metabolism, TCA cycle, and the respiratory electron transport (Figure 1F and Supplementary Figure S1E). These data suggest that in vitro-generated PSC-derived iPax3 myogenic progenitors have a predominant oxidative phosphorylation and glycolytic metabolic state, opposed to the predominant fatty acid-based metabolic state observed in adult satellite cells.

To understand how the in vivo environment might influence the transcriptional signature of these metabolic pathways, we performed similar analyses using published datasets of re-isolated iPax3 donor-derived satellite cells [12] (hereafter referred to as in vivo iPax3). PCA comparing in vivo iPax3 to adult satellite cells showed that these two cell populations shared a highly related signature (Figure 1G). Indeed, of the 360 DEGs found between in vitro iPax3 and satellite cells, only 3 (<1%) remained differentially expressed after exposure to the in vivo environment. This apparent overlap in the transcriptional profile between in vivo iPax3 and adult satellite cells prompted us to examine the signaling pathways previously identified as differentially expressed (Figure 1E,F). Here, we no longer found any statistical differences in expression of genes represented in metabolic pathways (Figure 1H,I, Supplementary Table S1). Using the adult satellite cells as the reference group, we looked at the fold change in gene expression for the top 50 DEGs in the in vitro iPax3 group compared to the in vivo iPax3 group (Supplementary Figure S1F). Here, we found that the relative gene expression for those genes changed towards zero after exposure to the in vivo environment, indicating a level of expression that is closer to the adult satellite cells. These data suggest that in vitro-generated iPax3 myogenic progenitors undergo transcriptional changes consistent with switching of metabolic pathways when exposed to the adult in vivo environment that are closely similar to that of bona fide satellite cells.

### 3.2. Metabolic Profile of In Vitro and In Vivo iPax3 Cells

**Figure 2:**
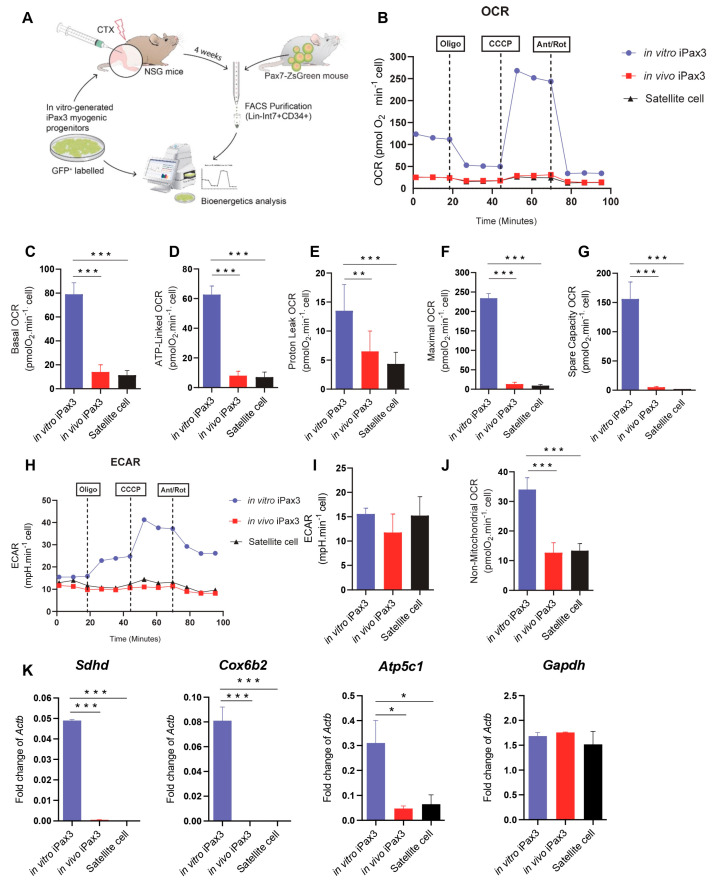
Bioenergetics of PSC-derived myogenic progenitors and re-isolated donor-derived satellite cells. (**A**) Outline of experiments. (**B**) Typical traces of real-time OCR before and after the addition of inhibitors to derive several parameters of mitochondrial respiration in Pax3 in vitro, iPax3 in vivo, and adult satellite cells (SC). (**C**) Initially, basal O_2_ consumption OCR was measured, from which basal cellular respiration can be derived by subtracting non-mitochondrial respiration. (**D**) ATP-linked represent the difference in OCR before and after Oligomycin. (**E**) H+ leak-linked OCR in Pax3 in vitro, iPax3 in vivo, and adult SC represent the difference in OCR after Oligomycin injection and Antimycin A and Rotenone. (**F**) Maximal OCR was determined via addition of a mitochondrial uncoupler CCCP that stimulates maximal respiration by mimicking a physiological energy demand, leading to an increase in oxygen consumption. (**G**) Spare respiratory capacity OCR reflects the difference between basal and maximal respiratory rate, and this capacity was determined by measuring OCR after treatment with oligomycin and CCCP. (**H**) Real-time whole-cell extracellular acidification rate (ECAR) is an indicator of the rate of acid efflux formed during glycolytic energy metabolism used to generate ATP. (**I**) ECAR quantification in iPax3 in vitro, iPax3 in vivo, and adult SC. (**J**) Non-mitochondrial respiration OCR has been observed at low levels in iPax3 in vivo and Adult SC. (**K**) Graphs shows gene expression for *Sdhd*, *Cox6b2*, *Atp5c1*, and *gapdh*. Results are normalized to Actb. Data are presented as mean ± SEM (*n* = three biological samples per group). * *p* < 0.05, ** *p* < 0.01, and *** *p* < 0.001 from one-way ANOVA followed by post hoc Tukey.

To determine whether the transcriptional switch of metabolic pathways is accompanied by functional changes, we next characterized the bioenergetic profiles of in vitro and in vivo iPax3 cells, as well as of bona fide adult satellite cells as a reference (Figure 2A). For these studies, we utilized in vitro-generated iPax3 myogenic progenitors, as previously described for transcriptomic studies [12]. For in vivo re-isolation, GFP-labelled myogenic progenitors were transplanted into pre-injured tibialis anterior (TA) muscles of immunocompromised NSG mice (Figure 2A). Upon confirmation of myofiber engraftment (Figure S2A), donor-derived satellite cells, identified as GFP+Lin-Int7+CD34+ [29], were purified (Figure S2B). Satellite cells were isolated from Pax7-ZsGreen mice [33] using ZsGreen in combination with the aforementioned satellite cell markers (Figure S2C). As expected, Pax7 expression was detected in bona fide satellite cells as well as in iPax3 donor-derived satellite cells, but not in iPax3 cells cultured in vitro (Figure S2D). We then examined these three cell populations for bioenergetics using the seahorse mitochondrial stress test, which allowed for measurements of oxygen consumption rate (OCR) and extracellular acidification rate (ECAR), indicators of aerobic respiration and glycolytic flux, respectively. This revealed that, under baseline conditions, in vitro iPax3 progenitor cells display enhanced OCR, but this decreases significantly upon engraftment as in vivo iPax3 cells show low rates of mitochondrial respiration, comparable to levels of adult satellite cells (Figure 2B,C). Similar findings were obtained with ATP-linked to OCR (Figure 2D). In addition, mitochondrial H^+^ (proton) leak, representing the remaining basal respiration not coupled to ATP, was high in in vitro iPax3 cells but decreased once these cells were exposed to the in vivo environment (Figure 2E). Likewise, the maximal oxygen consumption rate, measured by adding the uncoupler CCCP, was also higher in in vitro Pax3 cells when compared to in vivo iPax3 and satellite cells (Figure 2F). The spare respiratory capacity was elevated in in vitro iPax3 cells and much lower in both donor-derived iPax3 and bona fide satellite cell fractions (Figure 2G). However, the reserve capacity was clearly still present (Figure 2G and Supplementary Figure S2E), indicating that basal respiratory levels are not limited by overall respiratory capacity. 

The inhibition of mitochondrial respiration is often accompanied by an increase in fermentation of glucose to lactate and changes in concentration of hydrogen ions, which are accompanied by a drop in the pH levels. However, our data revealed that the ECAR in in vivo iPax3, as well as adult satellite cells (Figure 2H,I and Figure S2F), did not change, suggesting that purified skeletal muscle stem cell populations indeed have lower ATP production and do not undergo a shift towards acidification. Moreover, we observed that both these satellite cell fractions present lower rates of non-mitochondrial respiration (Figure 2J), suggesting that a subset of cellular enzymes continue to consume oxygen after addition of rotenone and antimycin A (Supplementary Figure S2E).

Gene expression studies confirmed these metabolic pathway preferences, as genes associated with mitochondrial oxidative phosphorylation, such as *Sdhd*, *Cox6b2*, and *Atp5c1*, were detected at high levels in in vitro iPax3 progenitors compared to in vivo iPax3 or adult satellite cells (Figure 2K). Meanwhile, there were no differences in the expression levels of *Gapdh*, a glycolytic enzyme (Figure 2K). These results further indicate that in vivo exposure to the adult muscle environment functionally modulates the mitochondrial oxygen consumption requirement of in vitro iPax3 myogenic progenitors.

### 3.3. Metabolomics Confirms Metabolic Switch of iPax3 Cells upon Engraftment

**Figure 3:**
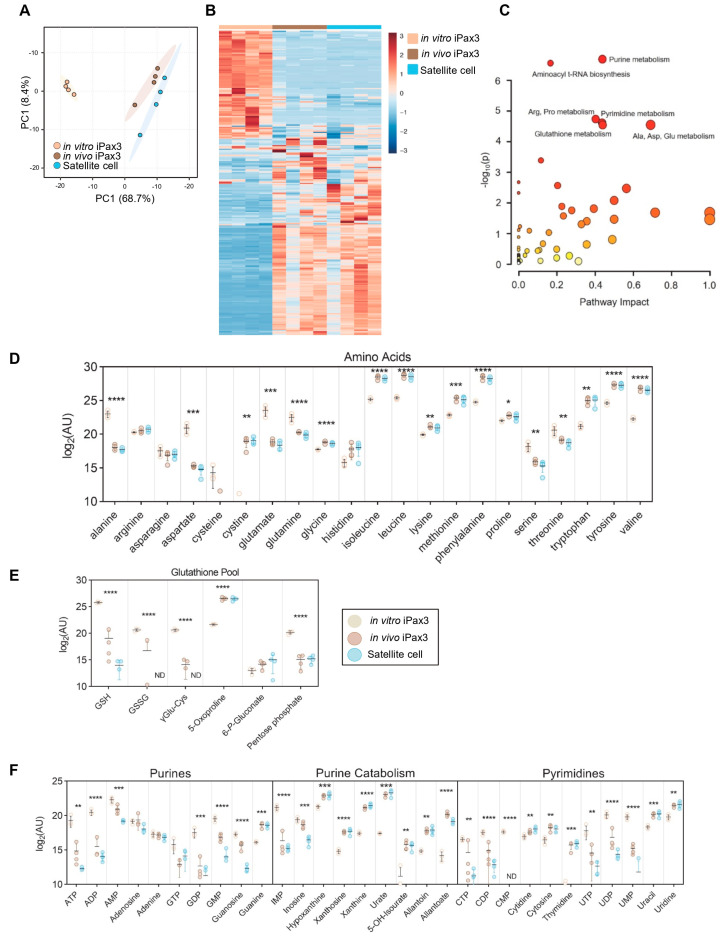
Metabolomics profiling of in vitro-generated iPax3 myogenic progenitors and post-transplant re-isolated iPax3 donor-derived satellite cells. (**A**) PCA of metabolomics data generated from in vitro iPax3 myogenic progenitors and re-isolated iPax3 donor-derived satellite cells (in vivo), along with satellite cells (*n* = four per group). (**B**) Hierarchical clustering analysis of these data color-coded from blue to red according to z-score. (**C**) Pathway enrichment analysis of ANOVA significant (*p* < 0.05) features. The size of each circle corresponds to its enrichment factor and color corresponds to *p*-value (from white to red). Individual values (log2[peak area in arbitrary units]) are shown for (**D**) amino acids, (**E**) glutathione, (GSH) homeostasis, and (**F**) purine and pyrimidine metabolism are shown. * *p* < 0.05, ** *p* < 0.01, *** *p* < 0.001, **** *p* < 0.0001, from two-way ANOVA comparisons.

To characterize steady state metabolic profiles of iPax3 cells before and after engraftment in comparison to adult satellite cells, mass spectrometry-based metabolomics was performed. Using accurate intact mass, isotopic pattern, fragmentation, and an in-house standard library, 250 metabolites were identified with quantification of relative abundance across the sample set. Unsupervised PCA confirmed significant clustering of each individual group, with in vivo iPax3 cells clustering near the adult satellite cell population (Figure 3A). Likewise, these cells shared multiple similarities in relative abundance of metabolites and were almost indistinguishable from satellite cells by hierarchical clustering analysis (Figure 3B, Supplementary Table S2). Some differences may be due to the distinctive regulation of gene expression by Pax3 and Pax7. When sorting for ANOVA significant metabolites, pathway enrichment analysis revealed that in vivo iPax3 cells most significantly mirror adult satellite cells in terms of purine metabolism, pyrimidine metabolism, amino acid metabolism, and glutathione homeostasis (Figure 3C). Indeed, the levels of many amino acids were significantly lower in both satellite cell fractions relative to in vitro iPax3 cells, including alanine, aspartate, glutamate, glutamine, serine, and threonine. Meanwhile, the levels of branched chain amino acids (leucine, isoleucine, and valine) and aromatic amino acids (phenylalanine, tryptophan, and tyrosine), as well as cystine, glycine, lysine, methionine, and proline, were all significantly elevated (Figure 3D). In terms of redox homeostasis, reduced (GSH) and oxidized (GSSG) glutathione were significantly reduced, while gamma-glutamyl cycle intermediate 5-oxoproline was significantly elevated (Figure 3E). Finally, the levels of purines and pyrimidines were significantly lower with observably higher levels of end-stage purine catabolites downstream from xanthine, indicating ongoing purine catabolism (Figure 3F).

### 3.4. iPax3 Cells Demonstrate Alterations to Energy Metabolism upon Engraftment

**Figure 4:**
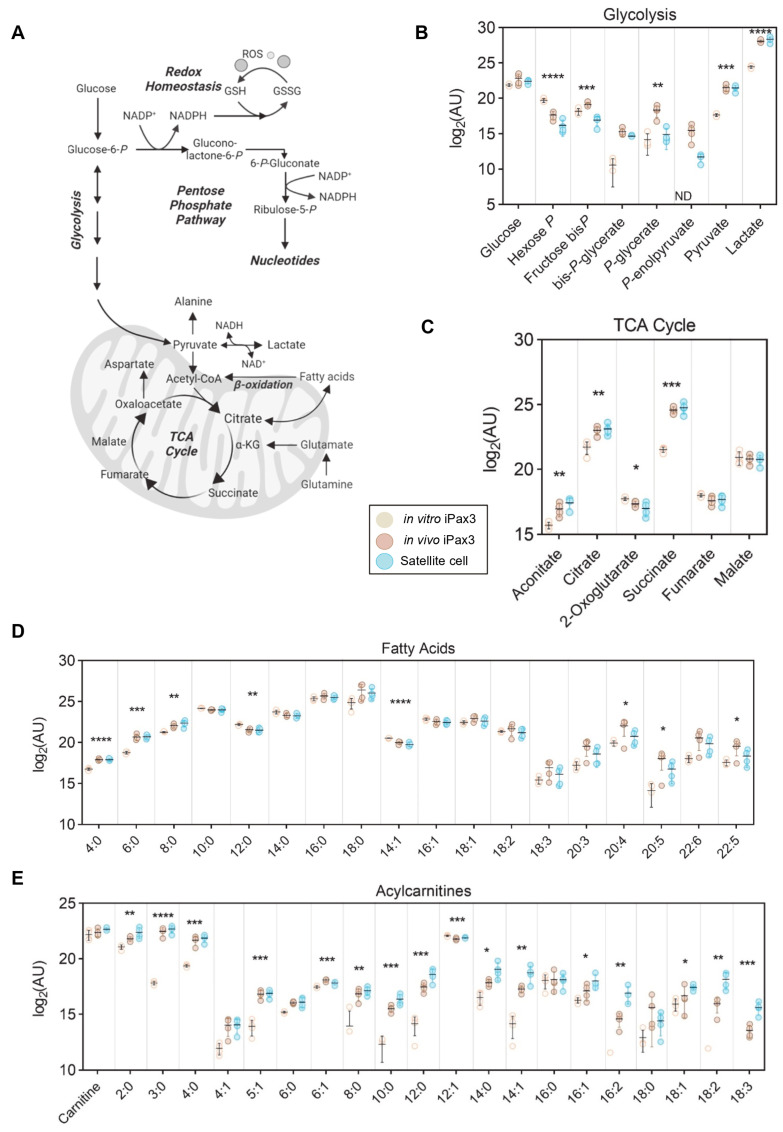
Energy metabolism of in vitro-generated iPax3 myogenic progenitors and re-isolated iPax3 donor-derived satellite cells. (**A**) A pathway map along with individual values (log2[peak area in arbitrary units]) are shown for (**B**) glycolysis, (**C**) tricarboxylic acid (TCA) cycle, (**D**) fatty acids, and (**E**) acylcarnitines. * *p* < 0.05, ** *p* < 0.01, *** *p* < 0.001, **** *p* < 0.0001, from two-way ANOVA comparisons.

The primary sources of energy in cells are derived through glycolysis and the TCA cycle (Figure 4A). Upon engraftment, iPax3 cells mirror the higher glycolytic rate of satellite cells, as demonstrated by decreased early-stage (glucose 6-phosphate) and increased late-stage (pyruvate and lactate) intermediates (Figure 4B). Despite a putative upregulation in glycolysis, these cells also demonstrate enhanced mitochondrial metabolism through significantly higher levels of TCA cycle intermediates aconitate, citrate, and succinate (Figure 4C). Considering the elevation and commitment to carbohydrate-fueled glycolysis, we then profiled the levels of free fatty acids, which also serve as a source of acetyl-CoA to fuel the TCA cycle. Both short (FA[4:0, 6:0, 8:0]) and long polyunsaturated (FA[20:4, 20:5, 22:5]) fatty acids were significantly higher, while medium-chain (FA[12:0, 14:1]) fatty acids were lower, indicating ongoing fatty acid oxidation (Figure 4D). Fatty acids are mobilized with coenzyme A (CoA) and transported to the mitochondria, at which point they are converted to acylcarnitines for transport across the mitochondrial membrane. In the mitochondrial matrix, they are converted back into acyl-CoA molecules for subsequent oxidation. As such, the levels of multiple acylcarnitines were significantly higher in both iPax3 and adult satellite cell fractions (Figure 4E). We then analyzed the metabolic pathways that had significantly changed in in vivo iPax3 compared to in vitro counterparts. Interestingly, the metabolism of glutamate, purine, arginine, proline, alanine, glycine, serine, glutathione and methionine, ammonia recycling, urea cycle, and phosphatidylcholine biosynthesis were the most modulated pathways in in vivo iPax3 cells (Supplementary Figure S3).

## 4. Discussion

There has been significant progress on the derivation of PSC lineage-specific cell types for the development of cell-based therapies for a number of diseases, including pigment epithelial sheet for the treatment of macular degeneration [34,35,36], dopaminergic neurons for the treatment of Parkinson’s disease [37,38,39], and islets for the treatment of type 1 diabetes [40,41], among others. In the case of skeletal muscle pathologies, such as muscular dystrophies, an effective cell therapy requires not only donor contribution to muscle fibers, but also to the satellite cell pool to ensure long-term regeneration. We and others have documented the presence of donor-derived myofibers and satellite cells upon the intramuscular transplantation of PSC-derived skeletal myogenic progenitors in mouse models of Duchenne muscular dystrophy [8,10,11,42,43,44,45,46], Limb-Girdle Muscular Dystrophy R9 [47,48], and Facioscapulohumeral muscular dystrophy [49]. Most of these studies defined satellite cell donor contribution based on Pax7 expression and localization under the basal lamina, but a few reports went further and validated the functionality of donor-derived satellite cells by performing re-injury and secondary transplantation assays [8,10,12,44]. In 2019, we reported that PSC-derived myogenic progenitors undergo significant transcriptomic changes upon exposure to the adult muscle environment and that the acquired postnatal molecular signature of donor-derived satellite cells correlates with enhanced engraftability as many fewer cells (20-fold) produce robust engraftment [12]. A recent publication by Sun and colleagues corroborates these findings [44]. Remarkably, both studies documented up-regulation of genes associated with extracellular matrix and notch signaling as well as the surface marker CD34 in re-isolated donor-derived satellite cells. While it has been indicated that CD34 expression identifies quiescent satellite cells [50], this marker alone may not be sufficient to conclude that PSC-derived myogenic progenitors give rise to quiescent satellite cells in vivo.

One biological process that clearly distinguishes quiescent (non-dividing) satellite cells from activated (proliferating) satellite cells is metabolic status. Quiescent satellite cells are characterized by low ATP levels and a distinctive metabolic phenotype that relies predominantly on fatty acid and pyruvate oxidization [17,51], which shifts towards increased glycolysis and glutaminolysis during satellite cell activation and proliferation [17]. Our analysis of datasets of post-transplant freshly isolated donor-derived satellite cells (in vivo iPax3) and bona fide satellite cells showed no statistical differences in gene expression profile of metabolic pathways between these two satellite cell fractions (Figure 1G–I). Meanwhile, in vitro iPax3 myogenic progenitors exhibited a very distinct profile from satellite cells (Figure 1D–F). These results are in agreement with our previous observations that post-transplant donor-derived Pax7^+^ satellite cells are non-cycling, as shown with Ki67 staining [8].

Bioenergetics and metabolomics studies provided similar findings, with in vivo iPax3 displaying comparable results to bona fide satellite cells (Figure 2, Figure 3 and Figure 4). Of note, all these experiments were performed side-by-side with satellite cells to make sure donor-derived and bona fide satellite cells were subjected to the same conditions prior to assessment of metabolic profile (24 h in culture). Broad scale steady state metabolomic assessments of these cell populations revealed similar trends in central energy pathways. Notably, in vivo iPax3 and bona fide satellite cells displayed similar upregulation of glycolysis as viewed through accumulation of end products pyruvate and lactate. Meanwhile, comparable phenotypes within mitochondrial pathways were observed, including accumulation of many TCA cycle intermediates. In addition to carbon input via glucose-derived pyruvate, similar relative trends in both fatty acids and acylcarnitines of varying acyl chain lengths indicated that these cell populations also maintain comparable fatty acid oxidation. Moreover, comparable profiles of purines and pyrimidines indicate that in vivo iPax3 cells mirror the anabolic and energetic charge state of satellite cells compared to in vitro iPax3 (Figure 4). An earlier study by Ryall and colleagues [17] demonstrated that quiescent satellite cells undergo an epigenetic-driven metabolic switch from fatty acid and pyruvate oxidation to glycolysis during transition to activation/proliferation. Our findings highlight a metabolic reprogramming in substrate utilization between in vitro and in vivo iPax3 cells, as indicated by the changes in energy production/nutrient utilization, and metabolic demand.

Pala and colleagues [16] have previously reported that during muscle growth, myogenic fetal cells rely mainly on glycolysis, whereas during early regeneration, activated satellite cells increase OxPhos and glycolysis. These same authors also documented that proliferating cells display higher ATP levels. As expected, in vitro iPax3 cells, which are highly proliferative, have higher ATP levels than donor-derived iPax3 and bona fide satellite cells. Glycolysis provides rapid generation of ATP as well as glycolytic intermediates that can be used for biosynthesis of nucleotides, lipids, and amino acids. Lower levels of purines, salvage intermediates, and pyrimidines observed here, in conjunction with lower levels of hexose phosphate (including glucose 6-phosphate) and higher lactate, indicate decreased utilization of the pentose phosphate pathway in both satellite cell fractions. In the absence of isotope tracing data, decreased steady state measurements of glutamine, glutamate, and transamination pair α-ketoglutarate along with higher levels of succinate suggest increased TCA cycle flux via anaplerosis. This reprogramming may also be evidenced by elevated levels of BCAA, which also provide TCA cycle carbon via succinyl-CoA and may additionally account for elevated levels of succinate. In accordance with the key role of fatty acid metabolism in defining quiescent satellite cells, it has been recently reported that dysregulation of lipid droplets (LP) biogenesis, whose function is to store extracellular fatty acids in the form of TAG, affects satellite cell homeostasis [52]. It has been suggested that that LP production may play an important role downstream of the PI3K/AKT/mTOR pathway.

As advances are made towards the realization of cell therapies for the treatment of muscle degenerative diseases, it is critical to understand the biophysical and metabolic changes that are inherent to the host and donor-derived biology. These data show that the metabolic adaptations of in vitro-generated PSC-derived myogenic progenitors to the in vivo environment make them almost indistinguishable from bona fide satellite cells.

## Data Availability

Data will be made available upon reasonable request. RNA-sequencing data was retrieved from published work using the Gene Expression Omnibus (GEO): GSE12161 and GSE121469.

## Supplementary Materials

The following supporting information can be downloaded at: https://www.mdpi.com/article/10.3390/cells13010076/s1, Figure S1: Transcriptional profile of in vitro-generated iPax3 myogenic progenitors (related to Figure 1). (A) Unsupervised hierarchical clustering of 360 metabolic DEGs between in vitro iPax3 and satellite cells. Gene ontology analysis of (B) Cluster 1 and (C) Cluster 2 from the heatmap shown in S1A. Clusters are indicated on the left margin. Gene-network plot of Reactome pathway enrichment analysis of the most (D) upregulated (Cluster 1) and (E) downregulated (Cluster 2) metabolic genes with each node scale on the top right and log_2_-fold change values on the bottom right. (F) Comparison of relative gene expression of the top 50 metabolic genes in in vitro iPax3 myogenic progenitors and in vivo iPax3 satellite cells with the red box indicating the reference range for adult satellite cells. Figure S2. Engraftment of iPax3 myogenic progenitors, donor-derived satellite cell isolation and mitochondrial OCR characterization. (A) Representative images show engraftment as indicated by the presence of donor-derived GFP^+^ (green) Dystrophin^+^ (purple) myofibers in TA muscles of NSG mice transplanted with iPax3 myogenic progenitors. DAPI stained nuclei (blue). Scale bar, 100 μM. (B) Representative FACS plots (upper panel) show gate settings for re-isolated iPax3 donor-derived satellite cells based on GFP^+^, lineage negative (Lin^−^), and CD34^+^/Int7^+^ expression. (C) Representative FACS plots (lower panel) show gate settings for freshly isolated adult satellite cells from the hind limb of 3-month-old Pax7-ZsGreen mice GFP^+^, lineage negative (Lin^−^), and CD34^+^/Int7^+^ expression. (D) Graphs shows Pax7 gene expression results for in vitro iPax3, in vivo iPax3, and adult SC normalized to Actb. (E) The rate of oxygen consumption (OCR) was an indicator of mitochondrial respiration, and (F) the rate of acid efflux or extracellular acidification rate (ECAR) of in vivo iPax3 and adult SC (ECAR) was shown. Data are presented as mean ± SEM of 3 independent experiments per condition. * *p* < 0.05 and *** *p* < 0.001 by one-way ANOVA followed by post-hoc Tukey. Figure S3. Metabolite pathway analysis and enrichment analysis. (A) Enrichment overview, and (B) Overview of enriched metabolite sets (top 25). Table S1: Gene expression for the top 300 metabolic DEGs in in vitro-generated iPax3 myogenic progenitors and in vivo iPax3 satellite cells. Table S2: Metabolomics datasets for in vitro iPax3, in vivo iPax3 and satellite cells.
