# Supplementary figures and images for: Metabolic Changes during In Vivo Maturation of PSC-Derived Skeletal Myogenic Progenitors
Source: Cells. 2023 Dec 29;13(1):76. doi: 10.3390/cells13010076 (PMC10778145; doi:10.3390/cells13010076)

**Figure S1**

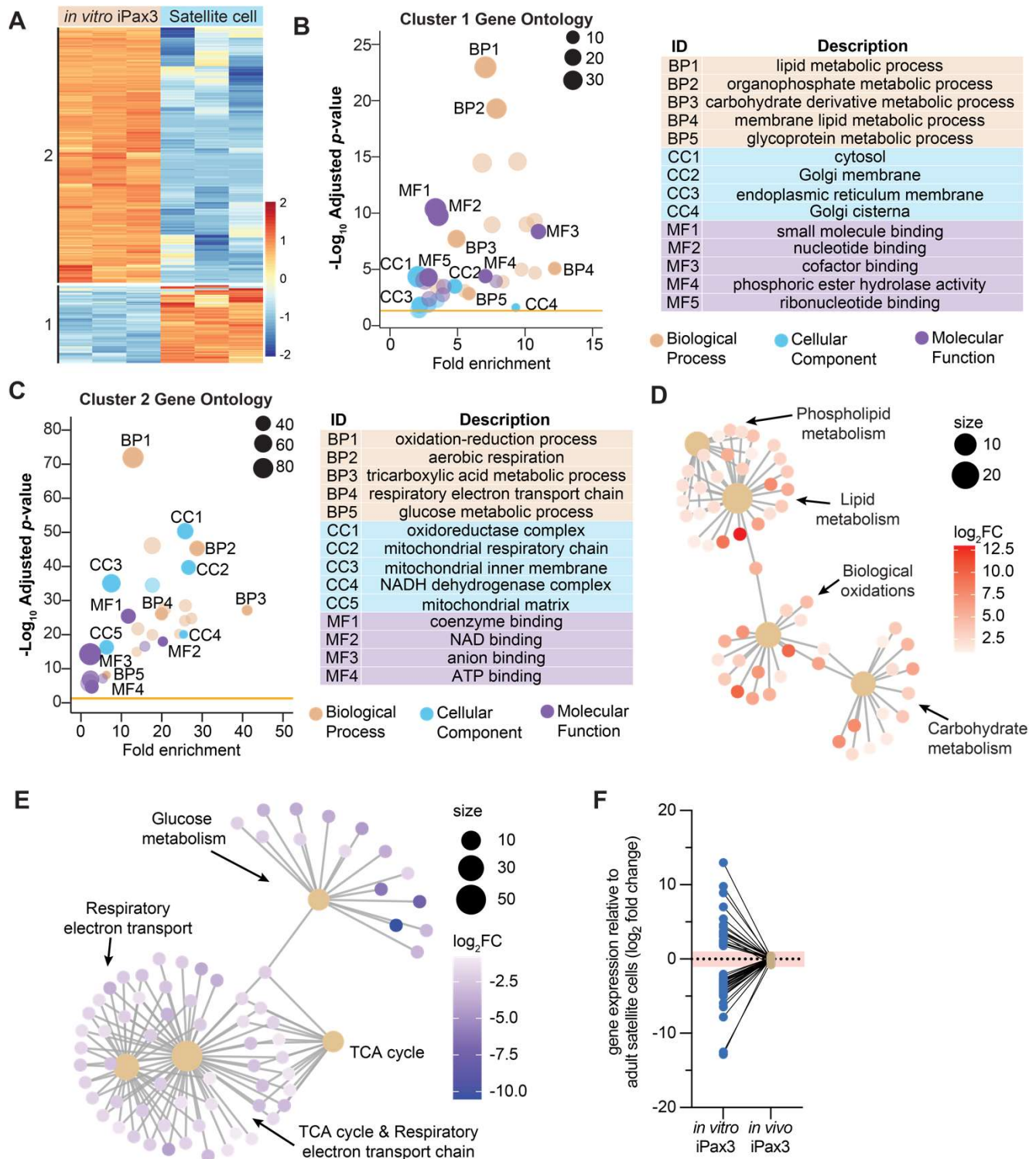

**Figure S2**

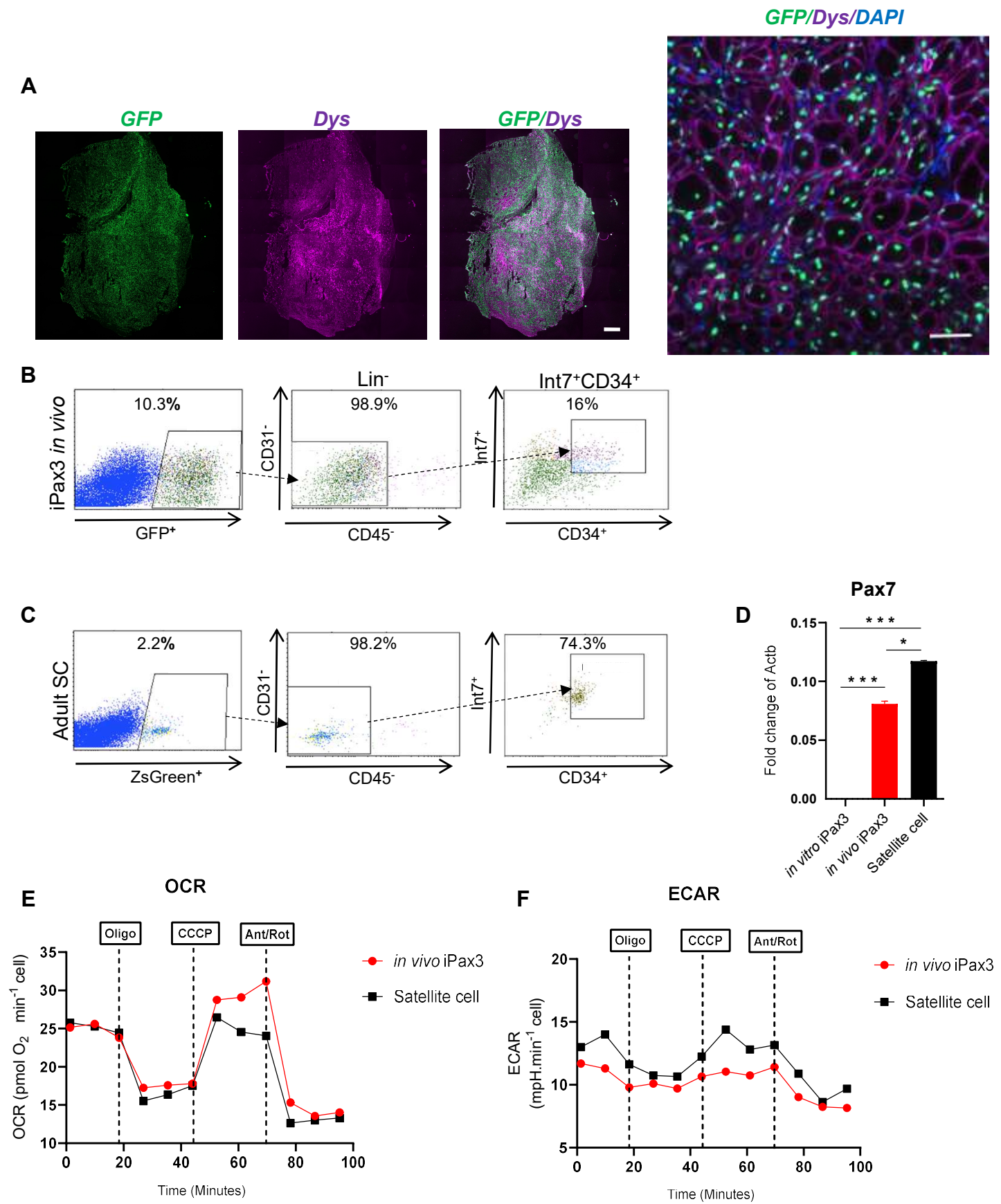

Figure S3

A

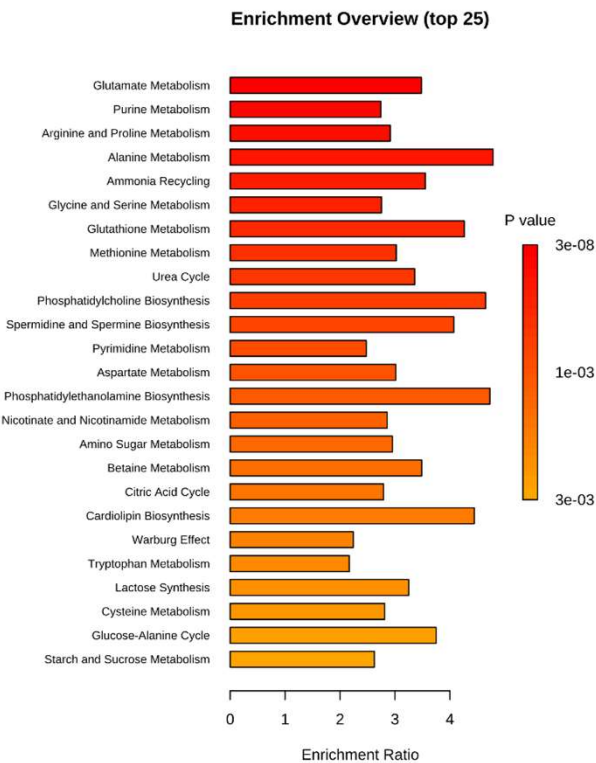

B

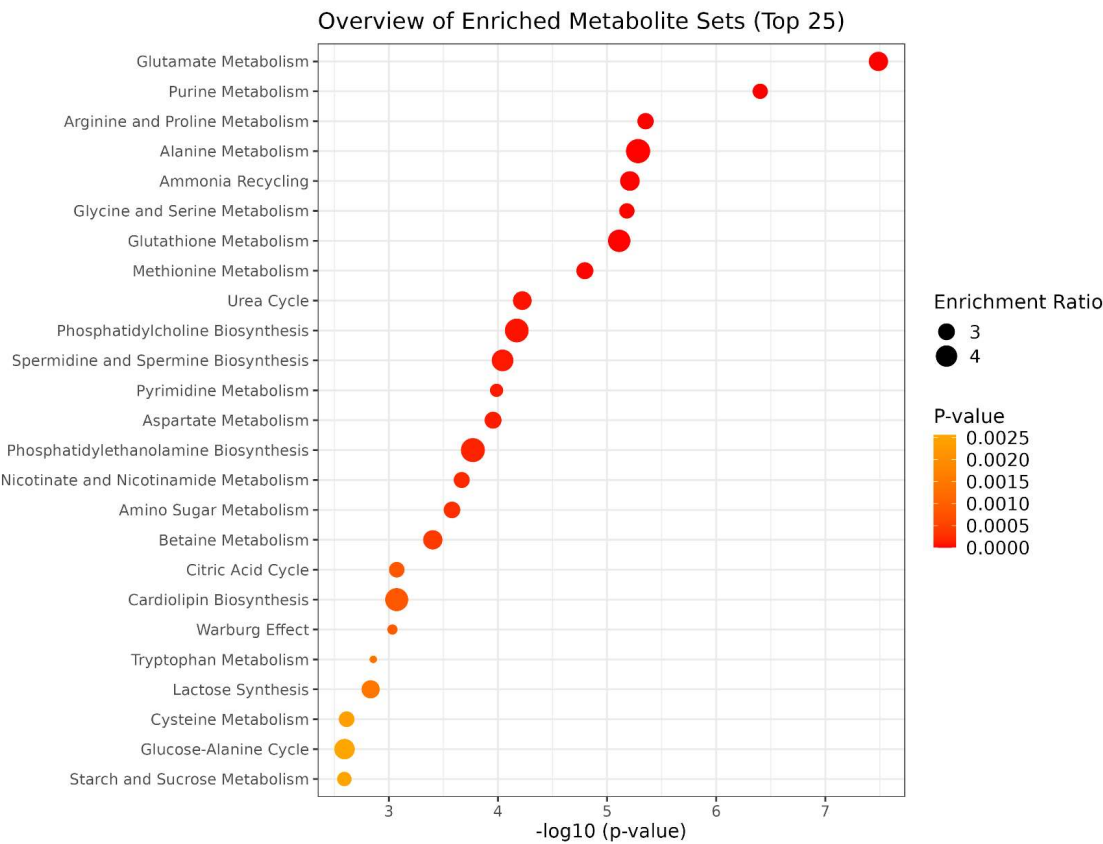

Supplement: Supplementary file 1 [file cells-13-00076-s001.zip › cells-2745972-Supplementary Materials/Supplementary Figures S1, S2, and S3.pdf]
